# Differential regulation of lineage-determining transcription factor expression in innate lymphoid cell and adaptive T helper cell subsets

**DOI:** 10.3389/fimmu.2022.1081153

**Published:** 2023-01-04

**Authors:** Difeng Fang, Ayanna Healy, Jinfang Zhu

**Affiliations:** Molecular and Cellular Immunoregulation Section, Laboratory of Immune System Biology, National Institute of Allergy and Infectious Diseases, National Institutes of Health, Bethesda, MD, United States

**Keywords:** lineage-determining transcription factor, epigenetic modification, CD4 T helper cells, innate lymphoid cells, Th cell differentiation, ILC development

## Abstract

CD4 T helper (Th) cell subsets, including Th1, Th2 and Th17 cells, and their innate counterparts innate lymphoid cell (ILC) subsets consisting of ILC1s, ILC2s and ILC3s, display similar effector cytokine-producing capabilities during pro-inflammatory immune responses. These lymphoid cell subsets utilize the same set of lineage-determining transcription factors (LDTFs) for their differentiation, development and functions. The distinct ontogeny and developmental niches between Th cells and ILCs indicate that they may adopt different external signals for the induction of LDTF during lineage commitment. Increasing evidence demonstrates that many conserved cis-regulatory elements at the gene loci of LDTFs are often preferentially utilized for the induction of LDTF expression during Th cell differentiation and ILC development at different stages. In this review, we discuss the functions of lineage-related cis-regulatory elements in inducing T-bet, GATA3 or RORγt expression based on the genetic evidence provided in recent publications. We also review and compare the upstream signals involved in LDTF induction in Th cells and ILCs both *in vitro* and *in vivo*. Finally, we discuss the possible mechanisms and physiological importance of regulating LDTF dynamic expression during ILC development and activation.

## Introduction

Innate lymphoid cells (ILCs) and adaptive CD4 T helper (Th) cells provide protection coordinately against pathogens and regulate tissue homeostasis, regeneration, and morphogenesis. Conventional effector Th cells are classified into multiple lineages, including interferon-gamma (IFN-γ)-producing type 1 Th (Th1) cells, interleukin-4 (IL-4)-, IL-5- and IL-13-producing Th2 cells, IL-17-producing Th17 cells, IL-21-producing follicular T helper (Tfh) cells, IL-9-producing Th9 cells, and IL-22-producing Th22 cells (1, 2). Lineage-determining transcription factors (LDTFs) T-bet (T-box expressed in T cells), GATA3 (GATA binding protein 3) and RORγt (retinoic acid-related orphan receptor gamma t) are critical for Th1, Th2 and Th17 cell differentiation and lineage maintenance, respectively (3–9). IFN-γ-producing type 1 ILCs (ILC1s) and natural killer (NK) cells belong to group 1 ILCs. ILC1s require T-bet for their development, whereas NK cells require both Eomes (Eomesodermin) and T-bet for their development and maturation (10, 11). IL-5- and IL-13-producing ILC2s depend on high levels of GATA3 for the lineage determination and development (12–14). RORγt is indispensable for the development of IL-17- and IL-22-producing ILC3s (15, 16).

Although ILC and Th cell subsets share some characteristics, they also exhibit different properties, including differences in ontologies, location, specificity and epigenetic modifications. Th cell differentiation occurs upon T cell receptor (TCR) engagement in pro-inflammatory cytokine environment. On the other hand, ILCs are pre-developed under homeostatic conditions and IL-7 signaling is required for their homeostasis. In addition, ILCs are not antigen-specific and thus develop independently of DNA recombination mediated by recombination-activating genes (RAGs) (17). Furthermore, ILCs are largely tissue-resident and rarely detected in peripheral secondary lymphoid organs or vascular system in steady state. Interestingly, most of the circulating NK cells do not express IL-7 receptor (IL-7R) but express inhibitory receptors, activating receptors and costimulatory receptors, which allow them to have certain levels of antigen-specific response in some conditions (18, 19). Genome-wide analyses of methylation and chromatin accessibility clearly show different epigenetic modification patterns between Th cell and ILC subsets (20–25). Although some cytokines that are involved in the induction of LDTF expression during Th cell differentiation can also upregulate LDTF expression in mature ILCs during their activation, these signals are usually dispensable for LDTF induction during ILC development (21, 26–28). Therefore, it is reasonable to assume that ILCs and Th cells could utilize different mechanisms for the initial induction of LDTF during their development and lineage commitment.

### Lineage-specific landscapes at the LDTF gene loci

To which extent the mechanisms for LDTF induction during Th cell differentiation and ILC development are mirrored or specified has not been well studied in the past. Epigenetic modifications are highly correlated with gene expression. For example, high levels of demethylation at the *Tbx21* and *Gata3* gene locus are detected in NK/ILC1s and ILC2s, respectively (20). Gene accessibility at the *Tbx21*, *Gata3* and *Rorc* loci in NK cells, ILC2s and ILC3s is also associated with their demethylation status (24). Recently, several studies have highlighted the lineage-specific landscapes at LDTF gene loci in Th cell and ILC subsets (21, 22, 29). In these studies, through CRISPR/Cas9-mediated genetic modification mouse models and *in vivo* disease models, genetic evidence has been provided to demonstrate the lineage-specific requirement of individual cis-regulatory element in response to external stimuli, including cytokines and cell-cell interactions, for the induction of LDTF in Th cells and ILCs.

### Cis-regulatory elements at the *Tbx21* locus in Th1 cells, NK cells and ILC1s

**Figure 1:**
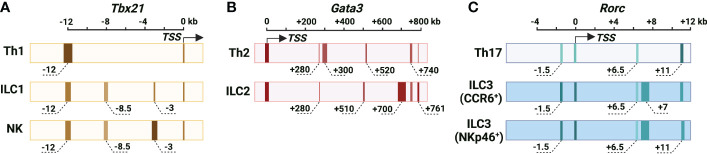
Cis-regulatory elements at the murine *Tbx21*, *Gata3* and *Rorc* gene loci. **(A)** The chromatin accessibility of the *Tbx21* gene locus in Th1 cells, NK cells and ILC1s. *Tbx21* promoter region is partially accessible in Th1 cells, NK cells and ILC1s. *Tbx21-CNS-12* is the major highly accessible site at the *Tbx21* gene locus in Th1 cells, and it contains STAT binding motifs and is essential for IL-12/STAT4- and IFN-γ/STAT1-mediated T-bet expression during Th1 cell differentiation. *Tbx21-CNS-12* is also a responsive element for T-bet expression during NK cell and ILC1 activation, but it is dispensable for T-bet induction during NK cell and ILC1 development. *Tbx21-CNS-3* is an NK-specific DHS, and it is critical for T-bet induction during NK cell development. *Tbx21-CNS-8.5* is an NK cell- and ILC1-specific DHS, but this region is dispensable for T-bet induction in NK cells and ILC1s. **(B)** The chromatin accessibility of the *Gata3* gene locus in Th2 cells and ILC2s. Long-range elements and promoter region are critical for the *Gata3* transcriptional activity. *Gata3-CNS+280* is highly accessible and important for GATA3 expression during thymic T cell and ILCP development, but it is only partially accessible and dispensable for the high levels of GATA3 expression in Th2 cells and ILC2s. The chromatin accessibility around *Gata3-CNS+700* increases as cells developing from ILCPs into ILC2Ps and ILC2s. The accessibility of *Gata3-CNS+700* is much higher in ILC2s than in Th2 cells, and this region is important for GATA3 expression in ILC2s and has a minimal effect on GATA3 expression in Th2 cells. *Gata3-CNS+761/762* has the *Gata3* enhancer activity in Th2 cells and ILC2s. *Gata3-CNS+740* is a Th2- and ILC2-specific DHS. *Gata3-CNS+300* and *Gata3-CNS+520* are Th2-specific DHSs. *Gata3-CNS+510* is an ILC2-specific DHS. **(C)** Chromatin accessibility of the *Rorc* gene locus in Th17 cells, CCR6^+^ ILCs and NKp46^+^ ILC3s. Overall chromatin accessibility at the *Rorc* gene locus in ILC3s is much higher than that in Th17 cells. *Rorc-CNS-1.5* and *Rorc-CNS+6* are important for epigenetic modifications of the whole *Rorc* gene locus associated with active chromatin and the induction of RORγt during Th17 cell differentiation. These two elements are accessible in CCR6^+^ ILC3s and NKp46^+^ ILC3s, but they have limited effect on ILC3 population in steady state. Whether they are responsive elements for RORγt expression upon ILC3 activation is unknown. *Rorc-CNS-11* is highly accessible in Th17 cells and ILC3s, and it is important for RORγt maintenance in Th17 cells. *Rorc-CNS-7* is an ILC3-specific element. The color from light to dark corresponds to chromatin accessibility from low to high. The width of elements stands for the width of peaks according to DHS-Seq or ATAC-Seq results. The approximate distance of the middle of peaks from TSS is labeled, and the “-” is for upstream and “+” is for downstream of TSS.

T-bet, a member of the T-box family of transcription factors, is encoded by the *Tbx21* gene and plays critical roles in Th1 cell lineage determination (5–7), ILC1 development and NK cell maturation (10, 11). Through DNase I hypersensitive site sequencing (DHS-Seq) and comparison of chromatin accessibility at the *Tbx21* gene locus in different ILC and Th cell subsets, several Th1-, NK- and ILC1-related DHSs have been identified (21) (**Figure 1**). While the chromatin accessibility at the promoter of *Tbx21* is comparable in Th1 cells, NK cells and ILC1s, the conserved non-coding sequence (CNS) 12 kb upstream of the *Tbx21* transcriptional start site (*Tbx21-CNS-12*) is highly accessible in Th1 cells, NK cells and ILC1s and contains STAT (signal transducer and activator of transcription) binding motifs. *Tbx21-CNS-12* is also accessible in naïve CD4 T cells indicating the readiness of this element to respond to cytokines even at the naïve cell stage. *Tbx21-CNS-12* is essential for T-bet induction during Th1 cell differentiation both *in vitro* and *in vivo*, but it is dispensable for the T-bet induction during NK cell and ILC1 development. By contrast, the element *Tbx21-CNS-3* is an NK-specific DHS with partial accessibility in ILC1s, but it is inaccessible in all Th cell subsets, ILC2s and ILC3s (21, 30). Accordingly, *Tbx21-CNS-3* is critical for optimal T-bet expression during NK cell development and thus for their maturation, but it has a minimal if any effect on T-bet expression in ILC1s and Th1 cells. Interestingly, while *Tbx21-CNS-8.5* is also an NK- and ILC1-specific DHS, this element is dispensable for T-bet expression neither in NK cells nor in ILC1s. Furthermore, the protein levels of T-bet in ILC1s from the *Tbx21-CNS-3* and *Tbx21-CNS-8.5* double knockout mice are still relatively normal indicating that a functional cis-regulatory element used by ILC1s for T-bet induction during their development is yet to be discovered, if it exists.

### T-bet induction in Th1 cells, NK cells and ILC1s

**Figure 2:**
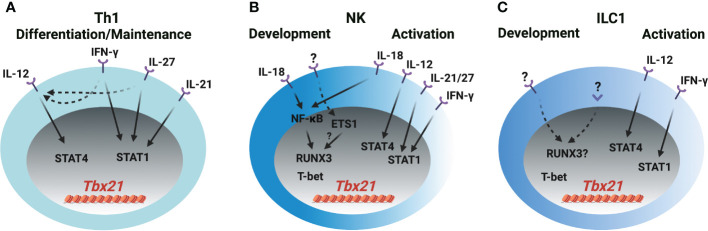
T-bet induction in Th1 cells, NK cells and ILC1s **(A)** IL-12/STAT4, IFN-γ/STAT1, IL-21/STAT1 and IL-27/STAT1 signaling induce T-bet expression through *Tbx21-CNS-12* during Th1 cell differentiation and maintenance. IFN-γ and IL-27 also upregulate IL-12R expression in T cells. **(B)** IL-18/NF-κB signaling induces RUNX3 expression and plays a role in the initial T-bet induction through *Tbx21-CNS-3* during NK cell development. ETS1 is critical for the T-bet expression in NKPs, pro-NK cells and mature NK cells. Whether ETS1 directly regulates T-bet induction or indirectly regulates T-bet induction through RUNX3 is not clear. T-bet may self-regulate its expression during NK cell development. IL-18/NF-κB/RUNX3 regulates T-bet expression through *Tbx21-CNS-3*, and IL-12/STAT4, IFN-γ/STAT1, IL-21/STAT1 and IL-27/STAT1 regulate T-bet expression through *Tbx21-CNS-12* during NK cell activation. **(C)** The mechanism of T-bet induction during ILC1 development is not clear. IL-12/STAT4 and IFN-γ/STAT1 may regulate T-bet expression during ILC1 activation. Only a small proportion of ILC1s express IL-18R.

Cytokines IL-12, IL-18, IL-21, IL-27 and IFN-γ are reported to be involved in T-bet expression during Th1 cell differentiation both *in vitro* and *in vivo*, however, it needs to be revisited whether the transcription factors activated by those cytokines directly induce T-bet expression or other secondary transcription factors that are induced by cytokines, in turn, regulate T-bet expression during Th1 cell differentiation. In addition, whether those cytokines are also involved in T-bet induction during NK cell and ILC1 development requires further investigation and discussion (**Figure 2**). IL-12 mediates the phosphorylation of STAT4 resulting in its binding to *Tbx21-CNS-12* in T cells and NK cells to upregulate T-bet expression (21, 26, 31–34). Abolishing STAT binding motifs at *Tbx21-CNS-12* or deleting STAT4 renders cells cultured under Th1 differentiation conditions incapable of expressing T-bet and effector cytokine IFN-γ (35, 36). However, mutating STAT binding motifs at *Tbx21-CNS-12* has no effect on T-bet expression in NK cells and ILC1s (21). Indeed, IL-12/STAT4 signaling is dispensable for T-bet induction during NK cell and ILC1 development in steady state; neither T-bet protein nor *Tbx21* mRNA are altered in *Stat4*-deficient NK cells and ILC1s, and neutralizing IL-12 doesn’t affect T-bet expression during NK cell development (21, 26).

Th1 cells, NK cells and ILC1s are professional IFN-γ-producing cells. The IFN-γ/STAT1 pathway is a potent inducer for *Tbx21* mRNA expression mainly through *Tbx21-CNS-12*, but such capability may be masked by the IL-12/STAT4 signaling as addition of exogenous IFN-γ in culture is unable to further upregulate T-bet expression at late stage of Th1 cell differentiation (21, 36–38). Nevertheless, *Tbx21-CNS-12* is readily accessible in naïve CD4 T cells, and IFN-γ can quickly induce *Tbx21* mRNA and subsequently *Il12rb2* mRNA in those cells (21, 37). Altogether, IFN-γ would be a critical player for the induction of T-bet during Th1 cell differentiation both *in vitro* and *in vivo*. Since *Tbx21-CNS-12* is highly accessible in NK cells and ILC1s, which also highly express IFN-γR (21, 39), it is plausible to anticipate an important role of IFN-γ in *Tbx21* mRNA induction during NK cell and ILC1 development and/or activation. T-bet expression is substantially reduced in the NK cells from the *Stat1*-deficient mice, but it is likely due to altered environmental niche rather than a cell intrinsic defect (40). Furthermore, the expression of T-bet by *Ifng*-deficient or *Tbx21-CNS-12*-mutant NK/ILC1s is intact (21, 26). Therefore, the IFN-γ/STAT1 pathway is dispensable for the initial induction of T-bet during NK cell and ILC1 development, although it may be involved in dynamic regulation of T-bet expression in activated NK cells and ILC1s.

IL-27 activates downstream STAT1 and STAT3 (41, 42), and quickly induces T-bet expression in the human T cell line (Jurkat), NK cell line (NKL) and naïve T cells (41). CD4 T cells incubated with IL-27 for more than twenty-four hours *in vitro* express high levels of T-bet (42–45). STAT1 is important for the optimal T-bet induction in the IL-27 supplemented Th1 polarization conditions, but IL-27 can still further upregulate T-bet expression in the absence of STAT1 (42). This may be due to the induction of IL-12Rβ2 and IFN-γ (41–44, 46). Since mature NK cells express the ligand-specific component of the IL-27R, WSX-1 (47), and *Tbx21-CNS-12* is accessible in NK cells and ILC1s, IL-27 may serve as an upstream signal for T-bet induction in these cells. Whether IL-27 signaling is involved in T-bet induction during early NK cell and ILC1 development needs further investigation.

IL-21 stimulation induces STAT1 binding to *Tbx21-CNS-12* and upregulates T-bet expression in CD4 T cells, but IL-21-mediated STAT3 activation represses T-bet upregulation (48). IL-21 is still able to upregulate T-bet expression in the *Tbx21-CNS-12* mutant mature NK cells, however, IL-21R is undetectable in the NK progenitors (NKPs) (21). A recent study shows that the effect of IL-18 on T-bet expression in T cells is minimal, and IL-18 alone doesn’t upregulate T-bet expression in CD4 T cells (21). However, IL-18 is capable of inducing IFN-γ expression in Th1 cells (21, 49) indicating that it may regulate T-bet expression through IFN-γ signaling pathway in CD4 T cells. On the other hand, IL-18 can upregulate T-bet expression in NK cells in a *Tbx21-CNS-12* independent manner. Furthermore, IL-18R is expressed in NK progenitors as early as at the refined NKP (rNKP) stage, and lower amounts of T-bet protein are detected in the *Il18r1*-deficient NK progenitors compared to their wild type counterparts (21). Thus, IL-18 signaling is involved in T-bet expression during both early NK cell development and late activation. However, IL-18 signaling pathway doesn’t induce *Tbx21* expression directly, instead RUNX3 induced by IL-18/NF-κB pathway promotes T-bet expression through *Tbx21-CNS-3* (21).

Anti-CD3 treatment quickly induces *Tbx21* mRNA in Th1 cell clones (5). *Tbx21* can be induced in the CD8-depleted *Stat4*-deficient lymph node cells under IL-12 and IL-18 neutralized T cell activation conditions after two days (50). However, TCR signaling alone is not able to induce T-bet expression in the *Tbx21-CNS-12* mutant CD4 T cells under Th1 conditions (21). Thus, the effect of auto-secreted IFN-γ on T-bet expression in the *in vitro* T cell activation system needs to be considered. Nevertheless, the T-bet expression in the Th1 cells from the early stage of Influenza- and Salmonella-infected mice is independent of IL-12, IFN-γ or IL-18 signaling (51), suggesting that the redundancy of these signaling pathways or other signals (e.g. TCR) may contribute to the initial T-bet induction.

Over-expression of RUNX3 promotes IFN-γ production, but RUNX3 is unable to upregulate T-bet expression in wild type Th1, *Tbx21-CNS-12* mutant “Th1”, or *Stat4* and *Ifngr1* double knockout “Th1” cells (21). Therefore, RUNX3 may not directly induce T-bet expression in CD4 T cells. T-bet expressing-ILC1s, NK cells and NKp46^+^ ILC3s are reduced in the NKp46-Cre- and PLZF-Cre-mediated *Cbfb*-deficient mice (52, 53). Yet, the protein levels of T-bet in the residual ILC1s, NK cells and NKp46^+^ ILC3s are relatively normal in the NKp46-Cre-mediated *Cbfb*-deficient cells (53). It is noticed that the expression of RUNX3 is earlier than T-bet induction during NK cell development (21, 53). According to the above observations, the functions of RUNXs/CBF-β on T-bet expression during innate cell development and maintenance require further investigation. ETS1 functions at early stages of NK cell development presumably through its binding to the *Tbx21* gene locus, and decreased levels of *Tbx21* mRNA is observed in the *Ets1*-deficient NKPs, pro-NK cells and mature NK (mNK) cells (54). Therefore, ETS1 may work as an important regulator for T-bet expression during NK cell development. However, ETS1 expression is reduced in the activated NK cells and NK cell lines compared with unstimulated mNK cells (54). In addition, ETS1 promotes RUNX3 expression in DP thymocytes (55), but whether ETS1 also regulates RUNX3 expression during ILC development is not known. Therefore, it will be interesting to further explore the upstream signals for the induction of RUNX family proteins and ETS1 during ILC development.

T-bet only binds to *Tbx21-CNS-12* in Th1 cells (21, 56), however, it binds to multiple elements, including *Tbx21-CNS-3*, *Tbx21-CNS-8.5* and *Tbx21-CNS-12*, in NK cells (21). Ectopic expression of T-bet promotes the endogenous expression of *Tbx21* under Th2 polarization conditions (50), but this autoactivation of T-bet may mainly be due to the secreted IFN-γ as such an effect is abrogated in the *Stat1*-deficient cells (38). Furthermore, the self-regulation of T-bet expression is only observed in the NK cells, but not in the Th1 cells by using the bacterial artificial chromosome (BAC)-transgenic T-bet reporter system (21, 57). Therefore, the binding of T-bet to *Tbx21-CNS-12* is not involved in regulating *Tbx21* expression in Th1 cells. Similarly, T-bet binds to *Tbx21-CNS-8.5* in NK cells, nevertheless, this element is dispensable for T-bet expression in NK cells at steady state (21). Finally, although NK cells and ILC1s are classified as two different lineages, it is difficult to define early ILC1 and NK progenitors, and thus it remains challenging to compare the initial mechanism of T-bet induction in ILC1 or NK progenitors.

### Cis-regulatory elements at the *Gata3* locus in Th2 cells and ILC2s

GATA3, encoded by gene *Gata3*, is required for the early T cell and ILC development, and the high levels of GATA3 are essential for Th2 cell differentiation and ILC2 lineage determination and development (3, 4, 12–14, 58–61). Different from the fact that the transcriptional activity of *Tbx21* and *Rorc* gene is regulated by the cis-regulatory elements near their promoter region, the expression of *Gata3* is largely controlled by the long-range elements in T cells and ILCs (22, 62, 63) (**Figure 1**). An element located at 280 kb downstream of the *Gata3* transcriptional start site (*Gata3-CNS+280*) is important for the GATA3 expression in thymic T cells and peripheral CD4 T cells by using BAC-transgenic strategy and CRISPR/Cas9-mediated knockout approach (22, 62, 63). *Gata3-CNS+280* deficiency also has a pan effect on ILC progenitors (ILCPs) and ILC subsets (22). However, this element seems dispensable for the high levels of GATA3 expression in Th2 cells and ILC2s (22, 62, 63). It is consistent with the observation that chromatin accessibility of *Gata3-CNS+280* is reduced in the mature Th2 cells and ILC2s comparing to naïve CD4 T cells and ILCPs by using assay for transposase-accessible chromatin using sequencing (ATAC-Seq) and DHS-Seq (21, 22). Together, *Gata3-CNS+280* is critical for the GATA3 expression at early stage of T cell and ILCP development, but it is not required for the high levels of GATA3 induction during Th2 cell differentiation and ILC2 lineage determination.

No ILC2- vs. Th2-specific chromatin accessible site at the large region around *Gata3-CNS+700* is found from ATAC-Seq data (22, 30). However, DHS-Seq result shows that the accessibility at *Gata3-CNS+700* region is much higher in ILC2s than that in Th2 cells and naïve CD4 T cells (21). Consistently, the deficiency of *Gata3-CNS+700* and nearby region results in a severer defect in GATA3 expression in ILC2s than in Th2 cells (22). The chromatin accessibility of *Gata3-CNS+700* region is increased as cells developing from ILCPs into ILC2Ps and then ILC2s, but this region remains closed in the other ILC subsets. The functions of ILC2s, but not of Group 1 ILCs and ILC3s, are greatly disturbed in the absence of *Gata3-CNS+700* and nearby region, indicating that this region has no effect on ILCP development (22). *Gata3-CNS+761/762* is considered as a type 2-specific enhancer through *H11 Gata3-CNS+761/762* EGPF reporter assay (22), but this site is only partially accessible in Th2 cells (21).

There are two Th2-specific DHSs located around *Gata3-CNS+300* and *Gata3-CNS+520*, one ILC2-specific DHS located around *Gata3-CNS+510*, and one Th2- and ILC2-specific DHS located around *Gata3-CNS+740* comparing to naïve CD4 T cells, other Th cell and ILC subsets (21). So far, the functions of those elements on GATA3 induction and Th2 cell differentiation and ILC2 development are still unknown.

### GATA3 induction in Th2 cells and ILC2s

**Figure 3:**
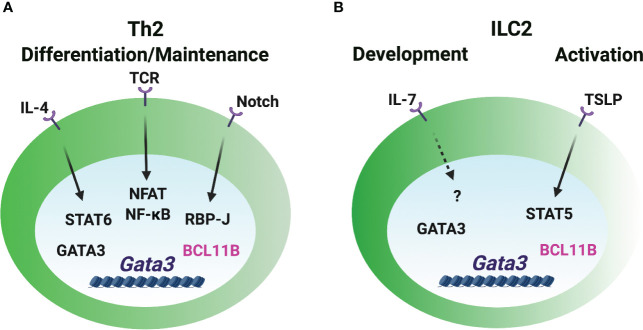
GATA3 induction in Th2 cells and ILC2s. **(A)** The *Gata3* promoters are critical for the GATA3 induction during Th2 cell differentiation. IL-4/STAT6 directly and vigorously induces GATA3 expression. Notch signal regulates *Gata3* promoter activity in CD4 T cells. TCR/NFAT/NF-κB signal is involved in GATA3 induction. GATA3 may self-regulate its expression in Th2 cells under certain circumstances. BCL11B works as a repressive regulator of GATA3 expression in already differentiated Th2 cells. **(B)** The booster of GATA3 induction during ILC2 lineage determination is unclear. IL-7 signaling may play a role in GATA3 expression in ILC2s. The release of repressive factor BCL11B may be important for the high levels of GATA3 expression. TSLP/STAT5 regulates GATA3 expression in the mature ILC2s, but it is dispensable for GATA3^hi^ ILC2 development.

IL-4 activates downstream STAT6 and vigorously induces GATA3 expression through distal and proximal promoters during naïve CD4 T cells differentiating into Th2 cells (64–67) (**Figure 3**). The binding of STAT6 to the responsive *Gata3* promoters is essential for the induction of GATA3 in T cells. STAT6 is critical for the reduction of repressive H3K27me3 modification and the increase of active H3K4me3 modification at the promoter region to induce *Gata3* expression (68, 69). IL-4 can upregulate GATA3 expression in the T cells which are not previously exposed to IL-4, but it fails to further upregulate GATA3 in already differentiated Th2 cells (66). Interestingly, *Stat6*-deficient IL-4-producing Th2 cells express intermediate levels of GATA3 (70). Therefore, an IL-4/STAT6-independent pathway may play a role in GATA3 induction in CD4 T cells. Although STAT5A and STAT5B can bind to the *Gata3* promoter, IL-2/STAT5 signaling likely works through IL-4 production to regulate GATA3 expression (71–74). The ILC2 population is intact in *Il4*/*Il13*-deficient mice (75), which indicates that IL-4 and IL-13 signaling is dispensable for the high levels of GATA3 expression in ILC2s. Similarly, GATA3 expression is also normal in the *Stat6*-deficient ILC2s (76), and IL-4/STAT6 only has a subtle effect on the activated GATA3^hi^ ILC2s from the parasite infected mice (77).

Certain strength of TCR signal induces early GATA3 expression (72). NFAT1, a TCR-inducible TF, binds to the *Gata3* promoters and several CNSs close to promoters, and it regulates GATA3 expression in a calcineurin-dependent manner (65). Furthermore, NF-κB plays a critical role in GATA3 induction during Th2 cell differentiation (78). RBP-J, a Notch effector molecule, binds to and induces the activity of the *Gata3* promoter in CD4 T cells (79). IL-2 receptor common γ chain (γc)-cytokine-mediated signaling seems to be dispensable for GATA3 expression in early ILC progenitors and their development (59), but IL-7 signal may be partially involved in the GATA3 expression during ILC2 development and activation *in vivo* (80). TSLP (thymic stromal lymphopoietin) activates STAT5 phosphorylation and induces GATA3 expression in the short-term cultured ILC2s (13). However, TSLP-TSLPR signal is dispensable for GATA3^hi^ ILC2 development and maintenance as it doesn’t affect GATA3 levels in ILC2s in steady state (14, 80).

Besides the importance of transcriptional activity of the *Gata3* promoters, long-distant cis-regulatory elements are also regulated by multiple TFs to induce GATA3 expression. Transcription factors TCF-1, HEB and CSL/RBP-J bind to *Gata3-CNS+280*. Transcription factors GATA3, CBF-β, RUNX1, RUNX3, GFI1 and BCL11B bind to *Gata3-CNS+761/762* in Th2 cells and ILC2s (22, 52, 69, 81–84), but they do not bind to *Gata3-CNS+280*. Whether those TFs are involved in GATA3 induction in Th2 cells and ILC2s is unclear. Repressive regulator, such as BCL11B, may play an essential role in controlling the levels of GATA3 during ILC2 and Th2 cell lineage determination. Deleting BCL11B upregulates GATA3 expression in ILC2s and Th2 cells *in vitro* (81, 85). Deletion of ETS1 results in a reduction of ILC2s in the bone marrow and mesenteric lymph nodes, but GATA3 expression is normal in the residual *Ets1*-deficient ILC2s (86). Since ETS1 is critical for the TF ID2 expression in the common helper-liker innate lymphoid progenitors (CHILPs) and ILC2s (86), ETS1 may not directly regulate GATA3 expression during ILC2 development. Ectopic expression of GATA3 promotes the endogenous *Gata3* induction under Th1- and Th2-polarization conditions in the absence of IL-4/STAT6 signaling (70). GATA3 binds to multiple *Gata3* cis-regulatory elements in Th2 cells and ILC2s (22, 81). *Gata3-CNS+761/762* bound by GATA3 in both Th2 cells and ILC2s exhibits *Gata3* enhancer activity (22). Th2-specific DHSs around *Gata3-CNS+300* are strongly bound by GATA3 (21, 81), yet, the activity of this element in Th2 cells has not been assessed. Taken together, the initial expression of GATA3 may be induced by TCR signaling in T cells and IL-7R signaling in ILCs through the promoter regions, and intracellular and extracellular signals triggered by these stimuli can further upregulate GATA3 expression. It is also possible that the release of repressive signal plays an important role in modulating the levels of GATA3, and GATA3 self-regulation may play a role in the maintenance of GATA3 levels under certain circumstances.

### Cis-regulatory elements at the *Rorc* locus in Th17 cells and ILC3s

The truncated form of the nuclear hormone receptor RORγ (RORγt), encoded by gene *Rorc*, is critical for Th17 cell differentiation and ILC3 lineage determination (8, 9, 15, 16). Strikingly, the overall chromatin accessibility at the *Rorc* gene locus in ILC3s is much higher than that in Th17 cells, and there are multiple DHSs at the *Rorc* gene locus in ILC3s (21) (**Figure 1**). A recent study shows that two cis-regulatory elements, *Rorc-CNS-1.5* (so called “CNS6” in the paper) and *Rorc-CNS+6.5* (so called “CNS9” in the paper) are important for RORγt induction during Th17 cell differentiation both *in vitro* and *in vivo* (29). Those two elements synergistically affect the optimal expression of RORγt in Th17 cells. Surprisingly, these elements do not function as enhancers, since neither *Rorc-CNS-1.5* nor *Rorc-CNS+6.5* promotes *Rorc* promoter activity in a dual luciferase reporter assay. Instead, they inhibit *Rorc* promoter activity. Nevertheless, they may play a critical role in the formation of DNA loops that facilitates enhancer-promoter interactions. *Rorc-CNS-1.5* and *Rorc-CNS+6.5* are important for active epigenetic modifications, such as H3Ac, H3K27Ac, H3K4me3 and RNA polymerase II (RNA Pol II) binding, at the whole *Rorc* gene locus in Th17 cells (29). These observations indicate that *Rorc-CNS-1.5* and *Rorc-CNS+6.5* are critical for the permissive structure of the *Rorc* gene locus in Th17 cells. *Rorc-CNS-11* is also highly accessible in Th17 cells and important for the maintenance of RORγt expression in Th17 cells (21, 29, 87).

Interestingly, although *Rorc-CNS-1.5* and *Rorc-CNS+6.5* are highly accessible in ILC3s, deleting *Rorc-CNS-1.5* or *Rorc-CNS+6.5* has a limited effect on ILC3 and thymic double-positive T cell population in steady state (21, 29). However, it is possible that these two elements will be utilized to regulate RORγt expression during ILC3 activation and thus their plasticity. While *Rorc-CNS-11* is accessible in both Th17 cells and ILC3s, *Rorc-CNS-7* is an ILC3-specific element (21). The functions of *Rorc-CNS-7* and *Rorc-CNS-11* on RORγt induction during ILC3 lineage determination require further investigation.

### RORγt induction in Th17 cells and ILC3s

**Figure 4:**
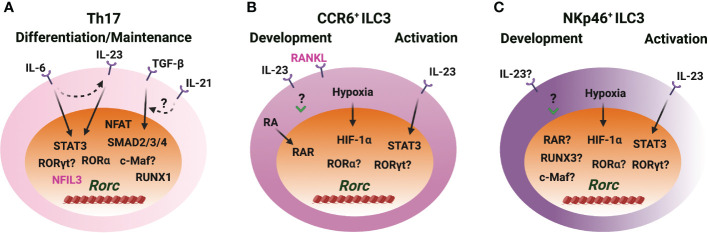
RORγt induction in Th17 cells and ILC3s. **(A)** IL-6/STAT3 and TGF-β/SMAD signaling induce RORγt expression through *Rorc-CNS-1.5* and *Rorc-CNS+6* during Th17 cell differentiation. IL-23/STAT3 plays a role in RORγt expression when IL-23R is upregulated upon naïve CD4 T cell activation. IL-21 synergizes with TGF-β to upregulate RORγt. RORα and RORγt bind to *Rorc-CNS-11* and play important roles in maintaining RORγt levels in Th17 cells. NFIL3 represses RORγt expression in CD4 T cells. **(B, C)** IL-23 upregulates RORγt expression during CCR6^+^ ILC3 development *in vitro*, but STAT3 is dispensable for RORγt expression during ILC3 development *in vivo*. IL-23/STAT3 signaling regulates RORγt expression in mature ILC3s. Hypoxia induced HIF-1α regulates RORγt expression in ILC3s. RA receptor RAR binds to the *Rorc* gene locus and regulates RORγt expression during CCR6^+^ ILC3 development and ILC3 activation. RUNX3 is critical for the RORγt expression in NKp46^+^ ILC3s. The role of RORα and RORγt in regulating RORγt expression in ILC3s is unclear. c-Maf benefits RORγt levels, but whether this is a direct effect is unknown. RANKL represses RORγt expression in CCR6^+^ ILC3s.

It is well accepted that cytokines IL-6 and TGF-β are essential for the initial induction of *Rorc* during Th17 cell differentiation (8, 9) (**Figure 4**). IL-6 stimulation induces the binding of STAT3 to *Rorc-CNS-1.5*, *Rorc-CNS+6.5* and *Rorc* promoter and changes the chromatin structure of the *Rorc* gene locus (23, 29, 88). *Rorc-CNS+6.5* is more important than *Rorc-CNS-1.5* in regulating chromatin changes of the *Rorc* gene locus and RORγt expression upon IL-6 engagement in CD4 T cells (29). The function of TGF-β on Th17 cell differentiation depends on other pro-inflammatory cytokines and the dose of TGF-β (89, 90). *Rorc-CNS-1.5* works as the main responsive element for TGF-β-induced RORγt expression and chromatin changes. TGF-β downstream transcription factors SMAD2/3, SMAD4 and c-MAF bind to *Rorc-CNS-1.5*, but not *Rorc-CNS+6.5*. Interestingly, the presence of *Rorc-CNS+6.5* is essential for the binding of TFs to *Rorc-CNS-1.5*, and *Rorc-CNS-1.5* affects STAT3 binding to *Rorc-CNS+6.5* (29). The synergistic effects of cis-regulatory elements *Rorc-CNS-1.5* and *Rorc-CNS+6.5* optimize the IL-6- and TGF-β-mediated RORγt expression and Th17 cell function.

IL-21 can synergize with TGF-β, but not with IL-6, to further upregulate RORγt expression during naïve CD4 T cell activation (91, 92). STAT3 directly binds to the *Il21* promoter and is critical for the production of IL-21 by Th17 cells (92). Thus, IL-21 works as an autocrine factor to promote RORγt expression and Th17 cell differentiation through STAT3 (91–94). Moreover, IL-6-induced IL-21 is independent of RORγt; IL-6 and IL-21 induce IL-23R expression in an RORγt-dependent manner; IL-23 upregulates *Rorc* expression in the IL-23R enforced-expressing CD4 T cells; TGF-β represses IL-6- and IL-21-mediated IL-23R upregulation (91). Altogether, the network and the feedback loops formed by IL-6, TGF-β, IL-21 and IL-23 signaling pathways control RORγt expression and Th17 cell differentiation and function.

Increasing evidence indicates that Th17 cells and ILC3s may also utilize distinct mechanisms for RORγt expression. Indeed, STAT3 is dispensable for RORγt expression during ILC3 development (95), although IL-23 stimulation *in vitro* significantly upregulates *Rorc* expression in the CD45^+^Lin^−^ leukocytes from the embryonic intestine on day E18.5 (96). Whether IL-6 or TGF-β plays a role in controlling high levels of RORγt upon ILC3 lineage activation is unknown. RUNX3, rather than RUNX1, is critical for RORγt expression in ILC3s (52, 53). Retrovirus-mediated over expression of RUNX3 fails to upregulate *Rorc* expression in Th17 cells (97). Over-expression of RUNX1 promotes *Rorc* expression and knockdown of RUNX1 reduces *Rorc* expression in Th17 cells (98). Luciferase activity assay also shows that RUNX1 promotes *Rorc* promoter activity while T-bet represses RUNX1-mediated *Rorc* promoter activity in Th17 cells (99). These studies indicate that RUNX1 is a positive regulator of *Rorc* during Th17 cell differentiation.

It is shown that c-Maf plays an important role in the balance between NKp46^+^ and CCR6^+^ ILC3s, and c-Maf harnesses T-bet expression and has a moderate effect on RORγt expression in NKp46^-^CCR6^-^ ILC3s (100, 101). c-Maf binds to *Rorc-CNS-1.5* in Th17 cells and in NKp46^+^ ILC3-like cell line MNK3 (29, 101). NFIL3, an important TF for early ILC development (102–105), directly binds to the *Rorc* promoter and negatively regulates RORγt expression in CD4 T cells (106). NFAT binds to the *Rorc* promoter and promotes RORγt expression in the activated CD4 T cells (107). RORγt expression is upregulated in ILC3s exposed to hypoxia *ex vivo* three hours in the presence of IL-1β and IL-23 and in MNK3 cells exposed to hypoxia without additional cytokines (108). Hypoxia induces HIF-1α binding to the *Rorc* gene locus in ILC3s (108). During Th17 cell differentiation, HIF-1α is upregulated in a STAT3-dependent manner, and HIF-1α binds to and activates *Rorc* luciferase reporter (109). Retinoic acid (RA), a metabolite of vitamin A, upregulates RORγt expression. RA receptors RAR and RXR can bind to the *Rorc* gene locus in CCR6^+^ ILC3s (110). Tumor necrosis factor (TNF) superfamily member receptor activator of nuclear factor κB ligand (RANKL) represses RORγt expression in CCR6^+^ ILC3s, but it has no effect in NKp46^+^ ILC3s and Th17 cells (111). RORγt and RORα bind to *Rorc-CNS-11* and maintain RORγt expression in Th17 cells (21, 29, 87). So far, many cues show that cytokines, physiological factors and stress are involved in RORγt expression during Th17 cell differentiation and ILC3 development.

## Conclusion and perspectives

Our previous understanding of LDTF induction during Th cell differentiation and ILC development is mainly based on the observations from *in vitro* studies. On one hand, it provides direct evidence to reveal the mechanisms of LDTF expression. On the other hand, regulatory network, feedback and space-time of multiple signals *in vivo* may be concealed. Seminal works on the differential requirements of Th cell subset- and ILC subset-specific cis-regulatory elements for LDTF induction in Th cells and ILCs provide a perspective to investigate the LDTF induction both *in vitro* and *in vivo*, as well as the cell development and functions (21, 22, 29).

Signals for LDTF induction during ILC development are less described in the past decade. Since ILCs that are not antigen-specific and may develop under sterile conditions, physiological factors or environmental stress may be involved in LDTF induction during ILC development. Endogenous factors, including mitochondrial reactive oxygen species (mROS), hypoxia, metabolites, products of lipid metabolism and complement, regulate sterile NLRP3 inflammasome priming and IL-1β and IL-18 production (112). IL-18 significantly induces RUNX3 expression, and RUNX3 plays multiple roles in controlling the development of several ILC subsets (21, 53). In addition, the factors mentioned above may be directly involved in LDTF expression through their downstream transcription factors. For example, hypoxia induces HIF-1α binding to the *Rorc* gene locus to upregulate RORγt expression in an ILC3 cell line (108). Notch signaling, a conserved developmental pathway, is also involved in ILC subset determination (113).

The induction of LDTF *in vivo* may differ from that *in vitro* depending on different properties of microbes and non-microbial substances. Although LDTFs antagonize each other (114), co-expression of two LDTFs are found in T cells and ILCs *in vivo*. T-bet-expressing pathogenic Th17 cells (115, 116), T-bet-, GATA3- or RORγt-expressing regulatory T (Treg) cells (89, 90, 117–119), and T-bet^+^RORγt^+^ ILC3s have been reported (120–122). Interestingly, during Treg cell development in the thymus (tTreg) and in the periphery (pTreg) as well as their maintenance in different tissues, distinct CNS elements are sequentially utilized for the induction and/or maintenance of Treg LDTF forkhead box P3 (Foxp3) expression (123–127). Therefore, it is likely that different CNS elements at the other LDTF loci may be preferentially used for fine-tuning the expression of these genes at different stages of T cell/ILC differentiation/development and activation. Furthermore, some cytokines, such as IFN-γ and IL-4, function as both LDTF drivers and the Th effector cytokines, which can result in a positive feedback loop for LDTF expression. In this review we focus on the positive regulators of LDTF expression, and it is possible that the release of repressive signals also plays critical roles in controlling LDTF expression. Therefore, the composition, concentration and time-space of cytokine environment together define the induction of LDTFs and cell differentiation/development.

Identifying Th cell subset- or ILC subset-specific cis-regulatory elements, evaluating the function of the elements on LDTF expression, finding TF binding motifs in the elements, and predicting upstream signals would be important strategies to reveal the mechanisms of LDTF induction during Th cell differentiation/maintenance and ILC development/activation. It should be noticed that some CNS cis-regulatory elements may code long noncoding RNAs (lncRNAs), which may regulate LDTF expression and cell development. Similarly, it will be important to analyze CNS elements at effector cytokine loci to determine cell type-specific regulation of these critical genes in T cell and ILC subsets. Finally, dissecting the developmental difference between ILCs and Th cells would help understand the space-time necessaries of innate ILCs and adaptive Th cells during immune responses, find new lineage-specific non-coding targets to engineer lymphocyte function, and discover novel interventions in treating immune diseases.

## Author contributions

DF prepared the first draft. AH provided critical comments and edited the manuscript. DF and JZ finalized the manuscript. All authors contributed to the article and approved the submitted version.
